# Supplementary material for: Severe urinary tract damage secondary to primary bladder neck obstruction in women
Source: PLoS One. 2021 Mar 19;16(3):e0248938. doi: 10.1371/journal.pone.0248938 (PMC7978344; doi:10.1371/journal.pone.0248938)
Supplement: Attachment — Submitted filename: Response to Reviewers final.doc [file pone.0248938.s002.doc]

January 31^st^, 2021

Israel Franco, M.D.

Academic Editor

PLOS ONE

PONE-D-20-39174
“Severe urinary tract damage secondary to primary bladder neck obstruction in women”

Dear Dr. Franco,

We are thankful for the opportunity to review and resubmit our manuscript and express our appreciation to the reviewers and the editor. We believe that their constructive comments and suggestions reflect a thorough analysis from experts with a solid scientific background and will significantly contribute to the quality of the study. Accordingly, we made changes to the manuscript and included our responses to reviewers’ comments.

Please find below the responses to the points raised by the reviewers.

RESPONSE TO THE REVIEWERS

**Reviewer: 1**

\# This is a retrospective review of case series including a small number of women (6) presenting primary bladder neck obstruction and treated by bladder neck incision at one institution.

ABSTRACT: Abstract is well written and points out the most important results.

INTRODUCTION: Introduction is fine. The authors report important findings previously described on the subject and point out the gap in the literature as well as their objective with their study

METHODS: I believe that due to the retrospective aspect of the study the authors may have found in their database review only the most severe cases, that needed BNI, which may be a bias to the study.

What were the reasons to choose one or two bladder neck incision?

**Reply:** As the reviewer cleverly noted, the fact that we were only able to identify patients that underwent BNI represents a bias in the study towards more severe cases. Such bias is stated in our original submission and remains in the 5^th^ and last paragraphs of DISCUSSION in this revised manuscript. The reviewer questioned the reasons for choosing between 1 and 2 incisions when performing BNI, which represents an important point in the surgical treatment of PBNO. Bladder neck incision has been performed with one[1], two[2,3], or even four[4] openings at variable sites ever since it was first proposed for female PBNO by Axelrod et al.[5]. Behind this heterogeneity lies a dilemma between adequate obstruction relief on the one hand versus the risk of serious (and possibly irreversible) incontinence on the other. So far, there appears to be no consensus on the best technique for BNI in women, yet the larger and more recent series [3,6,7] have used two incisions at the 4-5 and/or 7-8 o’clock positions. We favor a single incision either at the 4-5 or 7-8 o’clock positions. The second incision is performed when, based on intraoperative clinical judgment, the bladder neck does not seem to be open enough. A similar strategy was used by Grønbaek et al. [1]. We included this information in this revised manuscript. Our fear of complications from excessive incision seems not to be unjustified: in the largest series of BNI published up to date, Zhang et al.[7] reported incontinence and vesicovaginal fistula in 5% of their patients each. Accordingly, this step-up approach was intended to achieve what Dr. Jerry Blaivas described as “(…) is best to incise or resect “just enough” to relieve the obstruction, but not enough to cause sphincteric incontinence.” in his editorial comment for the same paper[7].

\# RESULTS: What do the authors attribute to be the reason for such a long period between the onset of symptoms and the diagnosis? It took more than 3 decades in one patient.

**Reply:** The long time between symptom onset and PBNO diagnosis also called our attention when reviewing our case series. Accordingly, we hypothesized (7th paragraph of the Discussion section) that it may be due to the uncharacteristic clinical presentation combined with the low frequency of the condition in the population, which require a high index of suspicion to be diagnosed. Moreover, the lack of knowledge about the condition in the medical (and even urological) community and the barriers imposed by an overloaded health system may have further delayed their referral to a specialized center.

\# Do the authors did EMG analysis during uroflowmetry or UDS? That could have shortened the time between the onset of symptoms and the diagnosis.

**Reply:** We do agree with the reviewer that EMG can add important information in the assessment of women with voiding problems, especially helping in the characterization of dysfunctional voiding. Unfortunately, we do not routinely perform EMG in adults in our urodynamic Lab. We use video-urodynamics - which is considered the gold standard test for the diagnosis of PBNO [8] - in all female patients when obstruction is suspected.

It is possible that the routine use of EMG could help speed the diagnosis of different causes of lower urinary tract dysfunction. However, it is important to clarify that whereas many patients had prolonged symptom duration, PBNO was diagnosed and treated within less than 6 months in all patients upon their first visit to our hospital. This underscores the importance of proper education on female PBNO to enable timely referral to tertiary centers before it results in severe damage to the urinary tract, as it was the case in our series.

\# Tables and figures are fine

DISCUSSION: The results on bladder outlet obstruction index (BOOI) should be described in the RESULTS section. Otherwise, discussion is fine.

**Reply:** We appreciate the insight and adjusted the manuscript in the way suggested by the reviewer. BOOI values were moved to RESULTS, whereas the contextualization of this finding with other series and how it relates to the hypotheses for our results was kept in DISCUSSION (modified fragments are highlighted in the text).

\# After reading the paper I would suggest the authors to review the title, since they included only severe bladder neck obstruction, and that is what the paper is about. It would be more accurate if the title was something like this: Urinary tract damage secondary to SEVERE primary bladder neck obstruction in women.

**Reply:** We understand the reviewer´s point of view but we feel that the current title is more attractive for the readers and more properly transmits the message we want to convey. Since both versions would be acceptable, we would be pleased to maintain the title as it is.

**Reviewer: 2**

Reviewer #2: The authors present a well written and compelling argument for pnbd.

As some one who sees many young patients and adolescents with this problem and believes that it is a problems that persists into adulthood it would have been beneficial if there was some data regarding these women and their symptoms as children or adolescents to tie this condition to a long standing problem to further be able to increase awareness of this insidious problem.

**Reply:** The reviewer highlights an important point since it is possible that PBNO appears in childhood or adolescence and progresses unnoticed until a late diagnosis in adulthood. Unfortunately, the retrospective nature of our study and the age of our patients (mean of 56.5 years; range 40 to 80 years) represent a major limitation in gathering reliable information on their voiding pattern as children or teenagers. Moreover, based on their symptom duration and age at diagnosis, one could hypothesize that PBNO appeared in adulthood. As discussed, however, the long history of LUTS combined with the insidious onset of the symptoms may in fact represent a condition that started decades before and presented due to inevitable decompensation in the lower (and upper) urinary tract. In this sense, the reviewer’s clinical observation provides an exciting topic for future research in the field, especially if these girls and adolescents are followed until they become mature adults.

\# if there are free flow stats on all patients it would be very useful to present this data as well so that the reader has a baseline from which to work prior to moving to invasive urodynamics. similarly what are the free flow numbers post incision.

**Reply: We acknowledge that data on free uroflowmetry could be useful for the characterization of this population. However, free uroflowmetry was available for only one patient because most women were either on clean intermittent catheterization or had a Foley catheter upon evaluation at our service.** The preoperative Qmax data that we present in this study were obtained from pressure-flow studies. **Postoperative free uroflowmetry was performed and the average results are presented in Table 4. Individual uroflow values may be disclosed to reviewers or via supplementary material if deemed necessary.**

\# We know from the pediatric literature that bn dysfunction is commonly associated with frontal lobe dysfunction and autonomic dysregulation. can you provide us with information regarding psychiatric diagnosis or disorders that the patients had and also if they were on any medications that would result in norepinephrine levels being elevated such as NERI, SSRI/NERI, sympathomimetics, Tricyclic antidepressants since these medications can exaggerate the response of the bn and create significant voiding issues.

**Reply:** We thank the reviewer for highlighting this very good issue, as there may be a link between bladder neck dysfunction, psychiatric conditions, autonomic dysfunction, and the use of sympathomimetic drugs could certainly impact on patients´ symptoms. This prompted us to review once more our database and we were not able to find neither psychiatric conditions nor the use of any drugs such as NERI, SSRI/NERI, sympathomimetics and/or Tricyclic antidepressants among our patients. Accordingly, we included a sentence to the manuscript in the METHODS section stating that this was evaluated and another in the RESULTS section underscoring this very important point.

**Reviewer: 3**

Reviewer #3: This is a report of women with severe bladder neck dysfunction who underwent bladder neck incision. The authors found this procedure to be effective for patients with severe lower urinary tract dysfunction secondary to PBNO. It is clinically useful to know that although PBNO can lead to severe bladder damage, BNI is an effective treatment.

\# is not clear in the introduction what is the authors' scientific hypothesis and research question. I understand that severe bladder changes have been poorly described for primary bladder neck dysfunction.

**Reply:** We are thankful for the valuable insight provided by the reviewer. Indeed, the scientific hypothesis and research question are the core elements of scientific communication. Ours is that female PBNO, so far mostly described as a source of bothersome LUTS, might include a spectrum of severe disease with significant damage to the bladder and upper tract. The six cases are then presented in detail to provide evidence for such hypothesis.

Accordingly, the last paragraph in INTRODUCTION was restructured (highlighted text) to express both the scientific hypothesis and research question in a clearer way.

\# However, the authors presented only two cystographic images with diverticulum and one MRI showing detrusor thickened. No image from the urodynamic study is presented. The CT image does not show thickened bladder. The hydronephrosis represented is mainly pelvis dilation.

**Reply:** The images play a central role in our study providing data on the damage features of these cases. We sought to present representative images of bladder and upper urinary tract damage, which had never been previously shown in the literature and represent novel information offered by our work. We opted to present different imaging modalities, including cystourethrograms, CT scans, and MRI. Figure 1A shows the characteristic appearance of the bladder neck obstruction during voiding with secondary damage to the bladder in the form of large diverticula. In figure 1B, from another patient, the bladder is massively distorted with multiple diverticula and secondary vesicoureteral reflux; Figure 1 C shows yet another patient with a massively thickened bladder wall at the MRI while figure 1 D is indeed a thickened bladder wall that appears attenuated by the overt bladder distension at the time of the exam.

Regarding the pressure-flow charts, our understanding is that the pressure-flow variables presented in Table 2 provide objective evidence of urodynamic obstruction for each patient. We chose not to include the urodynamic charts to avoid excessive images considering that our main focus was to characterize the secondary urinary tract damage in our population. The interested reader will find such charts in previous publications focused on the video-urodynamic diagnosis of PBNO in women [8, 9]. After carefully considering the reviewer’s feedback, we still hold the view that urodynamic tracings are not necessary in our study considering the present number of tables and images, but we would gladly include a pressure-flow chart should the reviewer and the editor find it helpful.

Please refer to our reply to the next question for our rebuttal regarding hydronephrosis.

\# As there are few cases presented by the authors it is important that more details of the cases are provided, such as the evolution of symptoms to urinary retention, history of urinary infection, urodynamics pictures and types of treatment before BNI.

The authors suggest hydronephrosis as an important finding of the study. However, in patients with urinary retention, bilateral renal dilation is the rule and not the exception. This is probably why this finding is not described extensively in the literature. Because it is an expected finding.

**Reply:** The reviewer suggested the inclusion of additional details regarding the evolution of symptoms to urinary retention, history of urinary infection, pressure-flow charts, and therapies before BNI. Although we agree that such information could be useful, we do not have more than what was included in the study. As mentioned before, the retrospective nature of our study represents a major limitation in gathering reliable information about the evolution of their symptoms through the past years. Patients typically presented to our tertiary hospital with severe damage to the bladder and/or upper urinary tract and most were already performing CIC or using a Foley catheter, which further hampers the ability to evaluate their symptoms before referral.

We respectfully disagree that “in patients with urinary retention, bilateral renal dilation is the rule and not the exception”. Bilateral hydronephrosis is found in a minority of patients with bladder outlet obstruction due to BPH, even among those with severe symptoms or on chronic catheterization [11]. It is a marker of end-stage bladder dysfunction and typically indicates the need for surgery (prostate resection, in patients with BPH) according to most guidelines[10]. In the context of adult female PBNO, further evidence that hydronephrosis is the exception rather than the rule comes from the two series in which it was reported: Peng et al. [3] found it in only 1 of 11 women who underwent BNI, whereas Zhang et al. [12] reported hydronephrosis in only 3 of 84 (3.5%) in the largest series to date. We highlight that these are surgical series with an expected bias towards more severe cases. Even so, hydronephrosis remained an uncommon finding.

Accordingly, Fig. 2 was intended to document upper tract dilation and its reversal after bladder drainage because it supports the core argument of the paper that PBNO comprises a spectrum of severe disease not appropriately described to date. Of note, hydronephrosis was found in 5/6 women (83%) in our series, contrasting to previous reports and supporting the above-mentioned hypothesis. In addition, four had hydronephrosis causing impaired renal function – including one patient who underwent kidney transplantation due to renal insufficiency caused by the PBNO - which underscores the significant risk for damage to the upper tract.

\# The authors comment that “Our findings, however, demonstrate that female PBNO may result in severe and irreversible damage to the bladder and the upper urinary tract.” However, although structural changes suggest severity, urodynamic is the most important finding for lower urinary tract evaluation. Video-urodynamic study is more important for the BNO diagnosis and for assessing vesicoureteral reflux than for diagnosing diverticulum, since its presence shouldn’t change the treatment.

**Reply:** We agree with the key role of UDN in the assessment of voiding dysfunction in women. Accordingly, we provide comprehensive data on the UDN of the 6 patients in Table 2. We also included (for the first time in a series of female PBNO) the Bladder outlet Obstruction Index of our patients, which was indeed very high. As discussed before, we opted not to include the UDN charts in the paper, instead opting to highlight the lower and upper urinary tract images that show the characteristic appearance of the bladder neck obstruction during voiding and secondary damage to the bladder and upper tract.

\# How did the maximum detrusor pressure evolve after the procedure?

**Reply:** We followed the patients based on symptoms, free uroflowmetry, postvoid residuals, renal function and upper tract radiological evaluation as needed. Although it would be interesting to have detrusor pressures postoperatively, we did not perform urodynamics as part of routine postoperative care.

\# Was sacral stigma a diagnosis of exclusion? How has spina bifida occulta been ruled out?

**Reply:** As part of the clinical assessment described in the 2nd paragraph of PATIENTS AND METHODS, the sacral area was examined (inspection and palpation) and was normal in all patients. None had symptoms or findings consistent with a neurological condition. In addition, sacral anomalies were not found in video-urodynamic images nor any other imaging that was obtained in the routine care of the patients.

\# In table 1 is creatinine at baseline or after CIC or after foley catheter placement? If it was at baseline, how many normalized with Foley?

**Reply:** Creatinine values in Table 1 refer to the most recent measurement at baseline (before bladder drainage with a Foley catheter or CIC). Creatinine levels returned to normal ranges in the 3 patients who had baseline elevated levels. This was informed in Table 4, which shows that at baseline 3/5 pts had impaired renal function compared to 0/5 patients postoperatively. One patient was removed from this analysis (as stated in the Table’s legend) because she was on hemodialysis and underwent kidney transplantation just before bladder neck incision**.**

\# How many used alpha blockers and for how long? Was standard urotherapy used and for how long? How about any type of electrical neuromodulation?

**Reply:** All patients in our series presented with urinary retention and severe damage to the bladder and/or upper tract. As detailed in the fourth paragraph of the RESULTS section, patient 5 was the only one who initially refused surgery and thus received a trial with doxazosin. She took the drug for 3 years but required ongoing CIC, eventually consenting for BNI after a kidney transplant with excellent results.

Since the diagnosis of bladder neck obstruction was clearly established for all patients, urotherapy and/or neuromodulation were not offered.

\# There is no indication where the discussion begins.

**Reply:** We followed the formatting model available at PLoS One website where ‘Results and discussion’ are placed together in the same section. Indeed, we agree with the reviewer and thus included a separate ‘Discussion’ section (highlighted in the text) for a clearer distinction between the two.

In the hope that we addressed the points raised the reviewers, we once again thank you for this opportunity.

Yours sincerely,

Cristiano M. Gomes, M.D.

Associate Professor

University of Sao Paulo School of Medicine – Department of Urology

Av. Dr. Eneas Carvalho de Aguiar, 255, Cerqueira César São Paulo, 05403-000, Brazil

crismgomes@uol.com.br

**References**

1.	Grønbaek K, Struckmann JR, Frimodt-Møller C. The treatment of female bladder neck dysfunction. Scand J Urol Nephrol. 1992;26(2):113-8.

2.	Blaivas JG, Flisser A, Tash JA. Treatment of primary bladder neck obstruction in women with transurethral resection of the bladder neck. J Urol. 2004 Mar;171(3):1172-5.

3.	Peng CH, Kuo HC. Transurethral incision of bladder neck in treatment of bladder neck obstruction in women. Urology. 2005 Feb;65(2):275-8.

4.	Jin XB, Qu HW, Liu H, et al. Modified transurethral incision for primary bladder neck obstruction in women: a method to improve voiding function without urinary incontinence. Urology. 2012 Feb;79(2):310-3.

5.	Axelrod SL, Blaivas JG. Bladder neck obstruction in women. J Urol. 1987 Mar;137(3):497-9.

6.	Markic D, Maricic A, Oguic R, et al. Transurethral bladder neck incision in women with primary bladder neck obstruction. Wien Klin Wochenschr. 2014 Apr;126(7-8):217-22.

7.	Zhang P, Wu ZJ, Xu L, et al. Bladder neck incision for female bladder neck obstruction: long-term outcomes. Urology. 2014 Apr;83(4):762-6.

8.	Brucker BM, Fong E, Shah S, et al. Urodynamic differences between dysfunctional voiding and primary bladder neck obstruction in women. Urology. 2012 Jul;80(1):55-60.

9.	Brucker BM, Nitti VW. Evaluation of Urinary Retention in Women: Pelvic Floor Dysfunction or Primary Bladder Neck Obstruction [ReviewPaper]. Current Bladder Dysfunction Reports. 2012 2012-06-30;7(3):222-229.

10.	Parsons JK, Barry MJ, Dahm P, et al. Surgical Management of Lower Urinary Tract	Symptoms Attributed to Beni gn Prostatic Hyperplasia: AUA GUIDELINES. 2020.

11. Sarmina I, Resnick MI. Obstructive uropathy in patients with benign prostatic hyperplasia. J Urol 1989;141:866–9.

12.	Zhang S, Zhang Q, Ji C, et al. Improved split renal function after percutaneous nephrostomy in young adults with severe hydronephrosis due to ureteropelvic junction obstruction. J Urol. 2015 Jan;193(1):191-5.
